# Looking Into Recent Suicide Rates and Trends in Malaysia: A Comparative Analysis

**DOI:** 10.3389/fpsyt.2021.770252

**Published:** 2022-01-05

**Authors:** Bob Lew, Kairi Kõlves, David Lester, Won Sun Chen, Nurashikin bt Ibrahim, Noor Raihan bt Khamal, Feisul Mustapha, Caryn Mei Hsien Chan, Norhayati Ibrahim, Ching Sin Siau, Lai Fong Chan

**Affiliations:** ^1^Australian Institute for Suicide Research and Prevention, School of Applied Psychology, Griffith University, Brisbane, QLD, Australia; ^2^World Health Organization Collaborating Centre for Research and Training in Suicide Prevention, Griffith University, Brisbane, QLD, Australia; ^3^Psychology Program, Stockton University, Galloway, NJ, United States; ^4^School of Health Sciences, Swinburne University of Technology, Melbourne, VIC, Australia; ^5^Non-communicable Diseases Section, Disease Control Division, Ministry of Health, Putrajaya, Malaysia; ^6^Centre for Community Health Studies (ReaCH), Faculty of Health Sciences, Universiti Kebangsaan Malaysia, Kuala Lumpur, Malaysia; ^7^Centre for Healthy Ageing and Wellness (H-Care), Faculty of Health Sciences, Universiti Kebangsaan Malaysia, Kuala Lumpur, Malaysia; ^8^Institute of Islam Hadhari, Universiti Kebangsaan Malaysia, Bangi, Malaysia; ^9^Department of Psychiatry, Faculty of Medicine, Universiti Kebangsaan Malaysia, Kuala Lumpur, Malaysia

**Keywords:** suicide rate, Malaysia, ASEAN, Muslim countries, G7, WHO Global Health Estimates

## Abstract

**Background:** Suicide is a preventable cause of death. Examining suicide rates and trends are important in shaping national suicide prevention strategies. Therefore, the objectives of this study were to analyze age-standardized suicide trends of Malaysia between 2000 and 2019 using the WHO Global Health Estimates data, and to compare the 2019 rate with countries from the Association of South-East Asian Nations (ASEAN), Muslim majority countries, and the Group of Seven (G7).

**Methods:** The age-standardized suicide rates data were extracted from the WHO Global Health Estimates. We calculated the average age-standardized suicide rates of the last 3 years from 2017 to 2019. Joinpoint regression analysis was conducted to calculate the average annual percentage change (APC) of the age-standardized suicide rates in Malaysia from 2000 to 2019.

**Results:** Between 2000 and 2019, the minimum and maximum suicide rates for both sexes in Malaysia were 4.9 and 6.1 per 100,000 population respectively, whilst the past 3-year (2017–2019) average rates were 5.6, 8.8, and 2.4 for both sexes, males, and females, respectively. The suicide rates decreased significantly for both sexes between 2000 and 2013. Between 2014 and 2019, the suicide rates increased significantly for males. In 2019, Malaysia recorded the rate of 5.8 per 100,000 population, with an estimated 1,841 suicide deaths, i.e., ~5 deaths per day. The Malaysian suicide rate was the second highest amongst selected Muslim majority countries, in the middle range amongst ASEAN countries, and lower than all G7 countries except Italy.

**Conclusions:** There is a need to further explore factors contributing to the higher suicide rates among Malaysian males. In light of the rising suicide rates in Malaysia, national mental health and suicide prevention initiatives are discussed and the importance of high-quality suicide surveillance data is emphasized.

## Introduction

Suicide remains a major public health problem for the world. The World Health Organization (WHO) estimates that there were over 700,000 deaths from suicide in the world in 2019, with an estimated suicide rate of 9.0 per 100,000 per year (1, 2). In addition, it is estimated that there may be anywhere from 10 to 20 attempted suicides for every suicide. Furthermore, each suicide leaves an estimated 135 other individuals exposed to the suicide (3). Reducing the global suicide mortality rate by one third by 2030 is a target (the only target for mental health) in the United Nations Sustainable Development Goals (SDGs) and in the WHO's Comprehensive Mental Health Action Plan 2013–2030 (4). WHO's 13th General Program of Work 2019–2023 includes the same indicator, aiming for a reduction of 15% by 2023 (5).

The countries of the world differ considerably in their suicide rate. Hungary had one of the highest suicide rates in the 20th century which, after the fall of the Soviet Union in 1989, were first published and the suicide rates in the Baltic and Slavic countries rose to the top (6, 7). Nevertheless, some nations have lower suicide rates, not necessarily because of poor reporting and recording of the cause of death. Lester (8) reviewed research on suicide in Muslim countries and found that the suicide rate was consistently low in countries where Islam was the predominant religion, and the protectiveness of Islam against suicide has been confirmed by other scholars (9–12). Therefore, the effect of poor reporting and recording of the cause of death, and the higher odds of undetermined, accidental, and other violent mortality rates as possible sources of underreported suicides, may be factors influencing the lower suicide rates reported in Muslim countries (13, 14).

National suicide rates vary over time. For example, suicide rates rose in Finland from 1950 (15.6) to 1975 (25.0), remaining steady after that until 1985 (15), showing a decline since 1990s (16). The rise in the suicide rate in Ireland over that time period (from 2.6 in 1950 to 7.8 in 1985), however, may have been partly the result of more accurate reporting and recording of suicides, even though longitudinal bias still needs to be accounted for Lester and Yang (15).

The recent update of the World Health Organization Global Health Estimates (1) includes suicide rates across the countries in 2000–2019. This provides an opportunity to analyze suicide trend in Malaysia and compare it with other countries. Previous reports of the suicide rates in Malaysia have reported a range from 3 to 13 per 100,000 population (17–19). A systematic review of studies published between 1972 and 2012 by Armitage et al. (20) presented an estimated suicide rate of 6–8 per 100,000 population per year, being higher among men, younger people, and the Indian minority.

The aim of the present paper is to analyze age-standardized suicide trends of Malaysia in 2000–2019 using WHO Global Health Estimates (1), and to compare the past 3-year (2017–2019) average rates in Malaysia (2019) with its neighboring countries in the Association of South-East Asian Nations (ASEAN), other Muslim majority countries, and high-income countries in the Group of Seven (G7).

## Materials and Methods

### Data Source

The suicide data used in this study for Malaysia are secondary data, extracted from the WHO Global Health Estimates (1). Age-standardized suicide rates were available for the period 2000–2019. The WHO Global Health Estimates of suicide mortality are currently the best quality international data available as they are utilizing relevant information from the Global Burden of Disease study and the WHO Mortality Database and as per their methodology consider potential contribution of undetermined deaths and accidents (2, 21).

### Comparison Groups

Three groups of countries were used for comparisons. The first group was countries belonging to the ASEAN, a regional grouping of 10 countries, i.e., Brunei, Cambodia, Indonesia, Laos, Malaysia, Myanmar, the Philippines, Singapore, Thailand, and Vietnam. Including these countries will provide important indicators of regional suicide rates for comparison. A second group of nine Muslim majority countries were derived based on having at least 50% of the population identifying as Muslim (22, 23), and being a part of the Middle East and North Africa (MENA) region. In addition, Indonesia and Pakistan were added to the group as they were Muslim majority countries in Asia with the largest and second largest global Muslim population, respectively (23). The third group comprised of high-income countries from the G7 organization, including Canada, France, Germany, Italy, Japan, the United Kingdom and the United States.

### Statistical Analysis

Joinpoint regression, also commonly known as change point regression or segmented regression, was used assuming the data can be separated into subsets—each with their own unique linear trend. This method is capable to identify the occurrence of statistically significant changes in trend, which defined as the values of the independent variable (*x*) where the slope of the linear function changes. The value of the change in trend may or may not be known prior to the analysis, it is generally unknown and must be estimated (24). Joinpoint regression provides an estimate of the (average) annual, monthly or weekly percentage change. In this study, the average annual percentage change (APC) was used to assess the annual change in the age-standardized suicide rates in Malaysia from 2000 to 2019, with 95% confidence intervals (95% CIs). A *p*-value of <0.05 was deemed statistically significant for all two-tailed tests. The analysis was conducted using Joinpoint, version 4.8.0. A breakpoint analysis was conducted using the R Segmented package to validate the results produced by the jointpoint regression. This analysis was performed using RStudio (25).

We calculated the average age-standardized suicide rates of the last 3 years from 2017 to 2019. The average ratios of age-standardized suicide rate of male to female were estimated. The average rate ratios in Malaysia in the last 3 years (2017–2019) were compared with its neighboring ASEAN countries, other Muslim majority countries, and high-income G7 countries.

## Results

**Figure 1:**
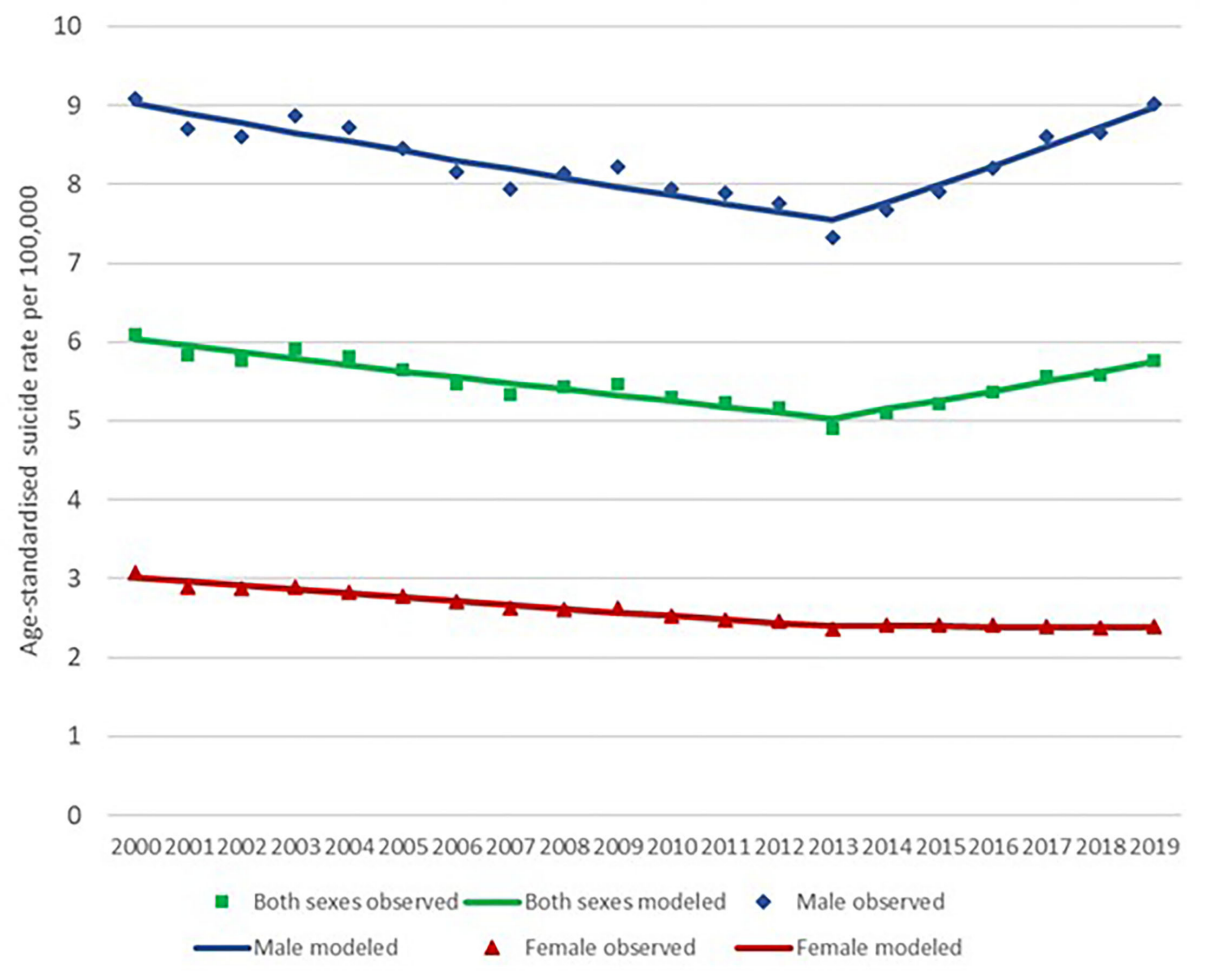
Malaysia suicide rate for 2000–2019 (for both sexes) with joinpoint analysis. Data sources: WHO Global Health Estimates (2021).

The age-standardized suicide rates in Malaysia between 2000 and 2019 ranged between 4.9 (2013) and 6.1 (2000) per 100,000 population. The average age-standardized suicide rate of the last 3 years from 2017 to 2019 in Malaysia was 5.6 per 100,000 population. There was a decrease of 19.7% in suicide rates between 2000 to 2013. Between 2014 and 2019, the suicide rates increased 17.8% from 4.90 to 5.77. The male to female ratio was between 2.9 (2000) and 3.8 (2019). The average male to female ratio of the last 3 years (2017 to 2019) was 3.7. Joinpoint regression identified 2013 as the joinpoint for both males and females (Figure 1). Age-standardized suicide rates declined significantly for males (APC: −1.4, 95%CI: −1.6 to −1.1) and females (APC: −1.7, 95%CI: −1.9 to −1.6) until 2013, which was followed by a significant increase for males (APC: 3.0, 95%CI: 2.0–3.9), but female rates remained stable (APC: −0.1, 95%CI: −0.6 to 0.5) (Tables 1, 2). The results from the breakpoint analysis have confirmed only one joinpoint existed in year 2013 for data with both sexes combined, male only data, and female only data, respectively.

**Table 1:** Age-standardized suicide rates during the past 20 years (2000–2019).

**Year**	**Age-standardized suicide rates (per 100,000 population)**			**Male: female ratio**
	**Both sexes**	**Male**	**Female**	
2000	6.10	9.08	3.08	2.9
2001	5.83	8.71	2.90	3.0
2002	5.77	8.60	2.88	3.0
2003	5.91	8.86	2.90	3.1
2004	5.81	8.72	2.83	3.1
2005	5.65	8.45	2.77	3.1
2006	5.47	8.15	2.71	3.0
2007	5.33	7.94	2.62	3.0
2008	5.43	8.14	2.61	3.1
2009	5.47	8.22	2.62	3.1
2010	5.30	7.94	2.53	3.1
2011	5.24	7.89	2.47	3.2
2012	5.17	7.76	2.46	3.2
2013	4.90	7.33	2.36	3.1
2014	5.10	7.68	2.41	3.2
2015	5.22	7.90	2.41	3.3
2016	5.37	8.20	2.41	3.4
2017	5.57	8.60	2.40	3.6
2018	5.58	8.66	2.37	3.7
2019	5.77	9.02	2.39	3.8

*Data source: WHO Global Health Estimates (2021)*.

**Table 2:** Joinpoint analysis of suicide rates in Malaysia for 2000–2019.

**Sex**	**Segment**	**Lower endpoint**	**Upper endpoint**	**APC**	**Lower CI**	**Upper CI**	**Test statistic (*t*)**	**Prob > |*t|***
Both sexes	1	2000	2013	−1.4^*^	−1.6	−1.2	−12.3	<0.001
	2	2013	2019	2.2^*^	1.5	3.1	6.1	<0.001
Male	1	2000	2013	−1.4^*^	−1.6	−1.1	−10.4	<0.001
	2	2013	2019	3.0^*^	2	3.9	6.9	<0.001
Female	1	2000	2013	−1.7^*^	−1.9	−1.6	−22.7	<0.001
	2	2013	2019	−0.1	−0.6	0.5	−0.2	0.82

*APC, Annual percentage change; CI, Confidence intervals*.

* *Significant at p <0.001*.

**Figure 2:**
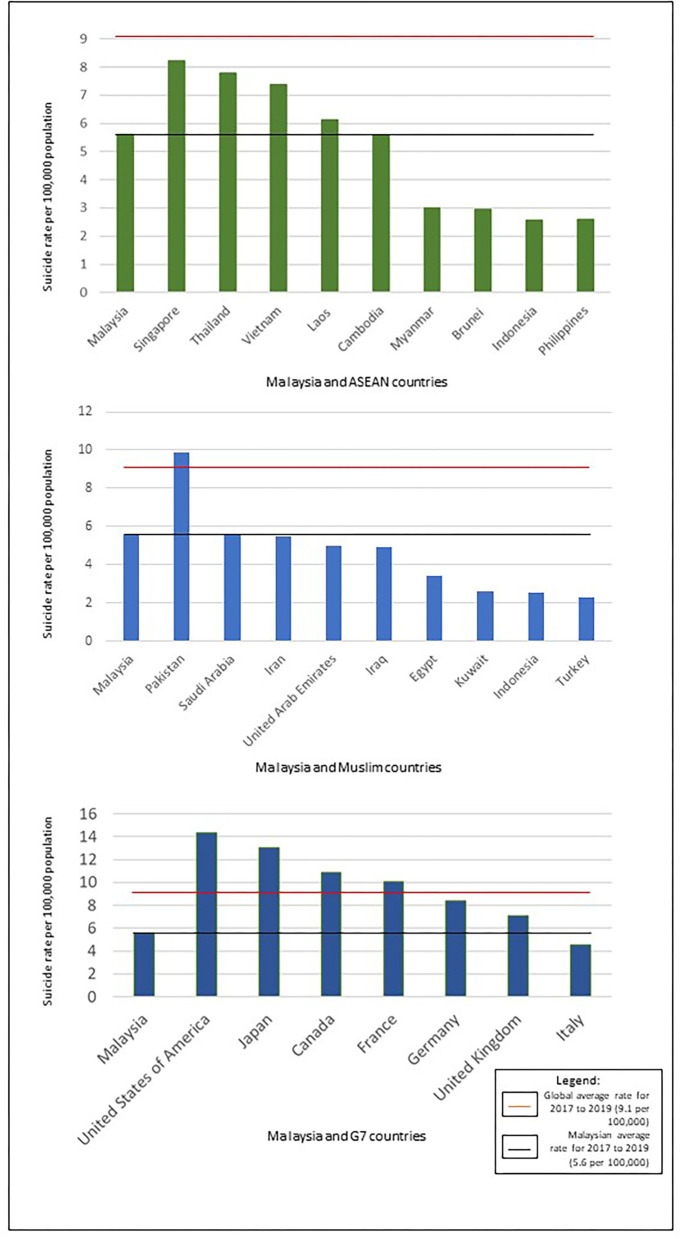
Comparisons of 3-year (2017–2018–2019) average suicide rates between Malaysia and other countries.

Table 3 compares the 3-year (2017–2019) average age-standardized suicide rate in Malaysia with other Muslim majority countries. Aside from Pakistan (which had the highest average suicide rate ratio when compared to Malaysia (rate ratio: 1.7), the total suicide rate in Malaysia was higher than the other Muslim majority countries (average rate ratio range of 0.4–1.0). Apart from Kuwait and Saudi Arabia, the male to female ratio was also the highest in Malaysia. On the other hand, the past 3-year average female rate of 2.4 per 100,000 population in Malaysia was lower compared to Pakistan, Iran, Iraq, and the United Arab Emirates (Table 3; Figure 2).

**Table 3:** Average age-standardized suicide rates during the past 3 years (2017–2019) of Malaysia and other selected Muslim-majority countries.

**Country**	**Age-standardized suicide rates (per 100,000 population)^§^**									**Past 3-year average (2017–2019)**			Average suicide rate ratio of Malaysia: other countries^*^			**Average male: female ratio**	**Muslim population(%) < /> 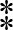 **
	**2017**			**2018**			**2019**										
	**Male**	**Female**	**Total**	**Male**	**Female**	**Total**	**Male**	**Female**	**Total**	**Male**	**Female**	**Total**	**Male**	**Female**	**Total**		
Malaysia	8.6	2.4	5.6	8.7	2.4	5.6	9.0	2.4	5.8	8.8	2.4	5.6				3.7	61.3
Pakistan	14.8	4.9	10.0	14.6	4.8	9.8	14.6	4.8	9.8	14.7	4.8	9.8	1.7	2.0	1.7	3.1	96.5
Saudi Arabia	8.2	2.1	5.7	8.0	2.0	5.6	7.8	1.9	5.4	8.0	2.0	5.6	0.9	0.8	1.0	4.0	97.1
Iran	8.6	3.0	5.8	8.1	2.9	5.5	7.5	2.8	5.1	8.1	2.9	5.5	0.9	1.2	1.0	2.8	76.0
UAE	5.7	2.6	4.8	6.0	2.6	5.0	6.3	2.6	5.2	6.0	2.6	5.0	0.7	1.1	0.9	2.3	99.4
Iraq	7.8	2.7	5.2	7.4	2.5	4.9	7.3	2.4	4.7	7.5	2.6	4.9	0.9	1.1	0.9	2.9	95.7
Egypt	4.7	2.2	3.4	4.6	2.2	3.4	4.7	2.2	3.4	4.6	2.2	3.4	0.5	0.9	0.6	2.1	92.4
Kuwait	3.6	0.7	2.5	3.7	0.7	2.6	3.8	0.7	2.7	3.7	0.7	2.6	0.4	0.3	0.5	5.1	74.6
Indonesia	4.0	1.2	2.6	4.0	1.2	2.6	4.0	1.2	2.6	4.0	1.2	2.6	0.5	0.5	0.5	3.4	87.2
Turkey	3.4	1.2	2.3	3.4	1.2	2.3	3.6	1.2	2.3	3.5	1.2	2.3	0.4	0.5	0.4	2.9	99.2

* *Calculated by dividing age-standardized suicide rate of other countries with the age-standardized suicide rate of Malaysia. Data sources*:

§ *WHO Global Health Estimates (2021). 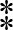
World Population Review (2021). UAE, United Arab Emirates*.

Table 4 compares the 3-year (2017 to 2019) average age-standardized suicide rates in Malaysia with other ASEAN countries. Malaysia had the fourth highest male, and fifth highest overall and female average age-standardized suicide rate among the 10 ASEAN countries. Singapore had the highest average suicide rate ratio compared to Malaysia (rate ratio: 1.5), while Indonesia, Brunei, Myanmar, and the Philippines had the lowest overall rate ratios (0.5) compared to Malaysia. Thailand had the highest rate ratio of male suicide rate (1.5) whilst Singapore had the highest rate ratio of female suicide rate (2.3) compared to Malaysia. On the other hand, Indonesia and the Philippines had the lowest rate ratios of male suicide rate (0.5) whilst Brunei had the lowest rate ratio of female suicide rate (0.4) compared to Malaysia. Thailand (5.6), Brunei (5.3), and Myanmar (4.6) had higher male to female ratios compared to Malaysia (Table 4; Figure 2).

**Table 4:** Average age-standardized suicide rates during the past 3 years (2017–2019) of Malaysia and other ASEAN countries.

**Country**	**Age-standardized suicide rates (per 100,000 population)^§^**									**Past 3-year average (2017–2019)**			Average suicide rate ratio of Malaysia: other countries^*^			**Average male:female ratio**
	**2017**			**2018**			**2019**					
	**Male**	**Female**	**Total**	**Male**	**Female**	**Total**	**Male**	**Female**	**Total**	**Male**	**Female**	**Total**	**Male**	**Female**	**Total**	
Malaysia	8.6	2.4	5.6	8.7	2.4	5.6	9.0	2.4	5.8	8.8	2.4	5.6				3.7
Singapore	9.5	5.1	7.3	10.4	5.0	7.8	12.7	6.4	9.7	10.8	5.5	8.2	1.2	2.3	1.5	2.0
Thailand	12.8	2.4	7.5	13.6	2.4	7.9	13.9	2.3	8.0	13.4	2.4	7.8	1.5	1.0	1.4	5.6
Vietnam	11.1	4.2	7.5	10.8	4.2	7.4	10.6	4.2	7.2	10.8	4.2	7.4	1.2	1.8	1.3	2.6
Laos	8.9	3.6	6.2	8.9	3.6	6.2	8.6	3.5	6.0	8.8	3.5	6.1	1.0	1.5	1.1	2.5
Cambodia	8.6	3.1	5.6	8.6	3.1	5.6	8.4	3.1	5.5	8.5	3.1	5.6	1.0	1.3	1.0	2.8
Myanmar	5.3	1.2	3.1	5.1	1.1	3.0	5.1	1.1	3.0	5.2	1.1	3.0	0.6	0.5	0.5	4.6
Brunei	5.4	1.0	3.3	4.9	1.0	3.0	4.2	0.8	2.5	4.8	0.9	2.9	0.6	0.4	0.5	5.3
Indonesia	4.0	1.2	2.6	4.0	1.2	2.6	4.0	1.2	2.6	4.0	1.2	2.6	0.5	0.5	0.5	3.4
Philippines	4.1	1.3	2.6	4.0	1.3	2.6	3.9	1.3	2.5	4.0	1.3	2.6	0.5	0.5	0.5	3.0

* *Calculated by dividing age-standardized suicide rate of other countries with the age-standardized suicide rate of Malaysia. Data sources*:

§ *WHO Global Health Estimates (2021). ASEAN, Association of South-East Asian Countries*.

Table 5 compares the 3-year (2017–2019) average age-standardized suicide rates in Malaysia with G7 countries. The average suicide rate in Malaysia was lower than all countries in the G7 group, except for Italy, which had the lowest rate ratio of suicide rate for both sexes (0.8), male (0.8), and female (0.9) compared to Malaysia. On the other hand, the US had the highest age-standardized suicide rate for both sexes and males, with the rate ratio of 2.5 for both sexes and males compared to Malaysia, whilst Japan had the highest female rate ratio of 3.1 compared to Malaysia. It is noteworthy that the male to female ratio of Malaysia was higher than in G7 countries (Table 5; Figure 2).

**Table 5:** Average age-standardized suicide rates during the past 3 years (2017–2019) of Malaysia and G7 countries.

**Country**	**Age-standardized suicide rates (per 100,000 population)^§^**									**Past 3-year average (2017–2019)**			Average suicide rate ratio of Malaysia: other countries^*^			**Average male:female ratio**
	**2017**			**2018**			**2019**					
	**Male**	**Female**	**Total**	**Male**	**Female**	**Total**	**Male**	**Female**	**Total**	**Male**	**Female**	**Total**	**Male**	**Female**	**Total**	
Malaysia	8.6	2.4	5.6	8.7	2.4	5.6	9.0	2.4	5.8	8.8	2.4	5.6				3.7
USA	22.5	6.5	14.4	21.8	6.6	14.1	22.4	6.8	14.5	22.2	6.6	14.3	2.5	2.8	2.5	3.4
Japan	19.4	7.6	13.5	18.9	7.9	13.4	17.5	6.9	12.2	18.6	7.5	13.0	2.1	3.1	2.3	2.5
Canada	17.6	5.8	11.7	15.6	5.5	10.5	15.4	5.4	10.3	16.2	5.5	10.8	1.8	2.3	1.9	2.9
France	16.4	4.9	10.4	15.8	4.7	10.1	15.2	4.5	9.7	15.8	4.7	10.0	1.8	2.0	1.8	3.3
Germany	12.9	3.9	8.3	13.1	4.0	8.5	12.8	3.9	8.3	13.0	3.9	8.4	1.5	1.7	1.5	3.3
United Kingdom	11.1	3.5	7.2	10.8	3.4	7.1	10.4	3.4	6.9	10.8	3.4	7.1	1.2	1.4	1.3	3.1
Italy	7.7	2.2	4.8	6.9	2.1	4.4	6.7	2.1	4.3	7.1	2.1	4.5	0.8	0.9	0.8	3.4

* *Calculated by dividing age-standardized suicide rate of other countries with the age-standardized suicide rate of Malaysia. Data sources*:

§ *WHO Global Health Estimates (2021). USA, United States of America*.

## Discussion

This study aimed to analyze the age-standardized suicide trends of Malaysia in 2000–2019 based on the WHO Global Health Estimates, and to compare the 3-year (2017–2019) average rates between Malaysia and ASEAN countries, selected Muslim countries, and G7 countries. It is important to note that the WHO GHE do consider potential misclassification of suicide under undetermined and accidental deaths on country level, therefore their rates are currently the most reliable suicide rates across WHO member states (21).

### The Suicide Rates in Malaysia

The estimated age-standardized suicide rate of 5.77 per 100,000 population in Malaysia was below the global average (9 per 100,000) in 2019. Past studies on suicide rates in Malaysia have shown consistently lower-than-average rates. For example, the systematic review of studies published between 1972 and 2012 by Armitage et al. (20) presented an estimated rate of between 6 and 8 per 100,000 population. However, the 2017–2019 average rate of 5.6 is notably higher than 1.18 per 100,000 population as reported by the National Suicide Registry Malaysia (NSRM) in 2009 (17). The differences may be attributed to differences in the methodology of data collection and calculation.

The collation of suicide data has been a challenge in Malaysia, as the source of suicide data is derived from a few sources which are the Malaysia Royal Police Department, Forensic Department of Ministry of Health, the Department of Statistics Malaysia, and the National Registration Department. The National Suicide Registry Malaysia was first developed in 2007 with the aim to create a nationwide system to capture data on death by suicide in Malaysia. Reports had been published for 4 consecutive years (2007–2010) (26). Currently, the Ministry of Health and relevant stakeholders are in the process of developing the National Suicide and Fatal Injury Registry Malaysia (NSFIRM) upon which suicide data will be accurately reported to obtain a national suicide rate figure for Malaysia. The NSFIRM is expected to be fully implemented by 2023.

On the other hand, the WHO estimated their data by “the extraction of codes X60–X84 and Y870 for suicide from the WHO Mortality Database, redistribution of deaths of unknown sex/age and deaths assigned to ill-defined codes, interpolation/extrapolation of number of deaths for missing years, scaling of total deaths by age and sex to WHO all-cause envelopes for 2000–2019, and use of population estimates from the UN Population Division” (1, p.2). As suggested by Maniam (18), taking into account deaths classified as “undetermined violent deaths” may be appropriate, as there may be a misclassification of suicide deaths due to the lack of evidence surrounding a death, or due to the pressure of societal stigma to underreport suicide deaths.

### The Suicide Trend in Malaysia

The suicide trend in Malaysia using Joinpoint regression showed a significant decline of age-standardized suicide rates for both sexes between 2000 and 2013, followed by a significant increase for males whilst female rates remained stable between 2014 and 2019. Nevertheless, those changes are rather hard to explain. A number of past studies have found an inverse association between economic growth and unemployment with suicide rates (27, 28). In Malaysia, the rise in suicide rates in 2014 seems to coincide with the drop in the Gross Domestic Product (GDP), which decreased 10.9% between 2014 and 2015, and dipped further in 2016, followed by an increase from 2017 onwards (29). The economic growth may have eased various strains within an individual's life, thus alleviating a significant amount of burden that may lead to suicide (28), while a lower GDP may contribute to unemployment which results in other life strains (27). The association between economic indicators and suicide rate warrants further investigation in Malaysia.

The increase in the suicide rates in Malaysia between 2014 and 2019 may be attributed largely to the increase in suicide among males. According to Alothman and Fogarty (30), a number of factors contributed to sex differences in suicide rates, including GDP per capita and gender development. The labor force in Malaysia is still predominantly male, with 80.8 and 55.6% labor force participation rate for males and females respectively in 2019 (31), and therefore males may have been more affected by negative economic indicators such as the GDP. Nevertheless, Malaysia ranked 61 globally in terms of Gender Development Index under the United Nations Development Program (UNDP) in year 2019 (32), and labor force participation had increased 8.4% from 2000 to 2019 (31). Studies have shown that as females are more empowered by development (e.g., education, labor force participation), their suicide rates have declined (30, 33). This may have further widened the sex-differences in suicide rates and warrants further investigation.

### Comparison of Suicide Rates in Malaysia With Other Countries

The 3-year (2017–2019) average suicide rate in Malaysia was the second highest when compared to other Muslim countries in the Middle East and Indonesia. This may be attributable to the multi-cultural composition of Malaysia including a variety of religions and ethnicities. Malaysia has the lowest proportion of Muslims (61.3%) compared to other Muslim majority countries in this study. In Malaysia, the suicide rate has been reported to be the highest among the Indian minority, followed by the Chinese (20), who are predominantly Hindu, Buddhists, and Christians. However, it is worth noting that all the Muslim majority countries registered lower suicide rates than the global average. This is consistent with previous studies that indicated the protectiveness of Islam against suicide among Muslims worldwide in general (34) and Malaysian Muslims in particular (35).

Compared with ASEAN countries, the Malaysian suicide rate ranked fifth for both sexes and females. The variation in the 3-year (2017–2019) average suicide rates of ASEAN countries, from 8.2 for Singapore to 2.6 per 100,000 population for Indonesia and the Philippines seems to be related to its varied demography in terms of economic development, religion, ethnicity, and history. Singapore as a high-income country (HIC) has the highest suicide rate than other countries in ASEAN, which were lower- to middle-income countries (LMICs). This is consistent with WHO report on suicide worldwide in 2019 noting that despite 77% of world suicides occur in LMICs, the suicide rates are higher in HICs (2). The difference in the suicide rate between HICs and LMICs is further reflected in comparison of Malaysia's suicide rate with G7 countries, as all G7 countries, except Italy, recorded higher suicide rates compared to Malaysia.

### Implications on Suicide Prevention Initiatives in Malaysia

The suicide trend in Malaysia is on the rise, in particular there is an increase in male suicides. Considering the suicide rate of 5.8 per 100,000 population in 2019, and given the population in Malaysia was 31.9 million in 2019 (36), there were approximately 1,841 suicide deaths in 2019, which equals to five deaths per day. This highlights importance of suicide prevention initiatives in Malaysia.

Suicide prevention initiatives in Malaysia started with the launch of a suicide helpline by The Befrienders Malaysia in the 1970s. In addition, the Ministry of Health (MOH) began their efforts to address mental health issues and suicide prevention through various activities, such as the World Suicide Prevention Day in 2004. The National Strategic and Action Plan for Suicide Prevention (2012–2016) was developed by the MOH in 2012 with the collaboration of WHO experts outlining main strategies, i.e., mental health promotion, early detection of mental health problems, capacity building of primary health care personnel on suicide prevention, as well as intersectoral collaboration with other agencies, monitoring and surveillance, and responsible media reporting (Mission Report Technical Support for the Development Including Training Materials, Disease Control Division in collaboration with WHO 2012). Another suicide prevention initiative was the Healthy Mind Programme launched in 2016 which was a collaboration between the MOH and the Ministry of Education (MOE) Malaysia that comprised mental health screening and suicide prevention training module of Teacher Counselors. The MOH has also collaborated with experts from universities to conduct postvention training sessions for individuals bereaved by suicide. These activities may have contributed to lowering the suicide rate and require further investigation.

Currently, the National Strategic Plan of Mental Health (2020–2025) has incorporated suicide prevention as one of the strategies in the plan. In 2019, a Technical Working Group on Suicide Prevention has been established consisting of experts from various stakeholders. The WHO has included Malaysia's suicide prevention strategy in the LIVE LIFE implementational guide for suicide prevention (37). Inter-ministerial and multi-agency collaboration have contributed to the upscaling of gatekeeper training for suicide prevention in the past decade. Frontline health workers, educators, and the general public have received training at regional and national levels from public-private local and international partnerships, as well as NGOs and patient advocacy groups. Siau et al. (38) reported improved awareness of suicide prevention in the short term amongst non-psychiatric health professionals. Future research is needed to build the evidence base for sustainable models of gatekeeper training that are culturally appropriate to diverse populations in Malaysia.

Malaysian media guidelines for safe media reporting of suicide were developed in 2004. However, Victor et al. (39) and Ng et al. (40) highlighted the lack of awareness and adherence to the guidelines. Current community-based safe messaging advocacy initiatives include capacity-building workshops, public forums and social media interactions by health professionals, people with lived experience, media professionals, academics and media industry regulatory bodies. Further evaluation of these safe messaging advocacy is warranted. Furthermore, it is important to note that pesticide poisoning is the second leading method of suicide in Malaysia (17). Findings from Leong et al.'s (41) study suggest that increased access to paraquat (a highly-lethal pesticide) may increase rates of paraquat poisoning. The impact of a recent national paraquat ban since 2020 on rates of fatal and non-fatal pesticide self-poisoning in Malaysia needs further investigation.

Efforts to decriminalize attempted suicide includes the amendment or repeal of Section 309 under the Penal Code which criminalizes attempted suicide as an offense. This is in line with the recommendations of the International Association for Suicide Prevention (IASP) and WHO to improve help seeking of individuals experiencing suicidal crisis. This in turn is hoped to eliminate society's stigma and lower the suicide mortality (42).

It is also important to note that surveillance of suicidal behaviors is essential for a public health model for effective suicide prevention (1). Maniam's (18) observation on the corresponding rise of undetermined violent deaths following the suicide rate reduction from 1975 to 1990 indicated that the misclassification of suicide deaths had occurred on a large scale, leading to the conclusion that the national suicide statistics were not reliable. Currently, Malaysia lacks a high-quality real-time surveillance system of suicidal behaviors. Such a system is essential for identifying the at-risk populations to tailor suicide prevention activities and evaluate their impact. This is more important than ever in the light of the current COVID-19 pandemic, which has led to much speculation regarding changes in suicide rates. Although, a recent analysis of mainly HIC showed no increase in suicides in 2020, authors called for the need to continue surveillance across the world (43).

### Limitations of the Study

This study used secondary data derived from the WHO Global Health Estimates. While there are causes of death which may have been underreported, the WHO GHE for mortality do consider that possibility and have utilized relevant information (e.g., undetermined causes of death, accidental deaths) in their country-specific estimates (21). Therefore, there is no reason to believe that changes in the presented trend are artefactual or that the information is not comparable across the countries or subject to major error.

We did not quantitatively investigate the possible factors influencing the suicide rates and trends. When comparing the rates of Malaysia with other countries, we acknowledge that there are many contextual reasons and interactions which prevents us from making any conclusions on the protectiveness of Islam or any other one factor. We also could not draw conclusions regarding the impact of the ongoing suicide prevention initiatives on the suicide rates.

## Conclusions

The mean age-standardized suicide rates in Malaysia between 2000 and 2019 was 5.5, and ranged between 4.9 (2013) and 6.1 (2000) per 100,000 population. The GHE data recorded a decrease in suicide rates for both sexes between 2000 and 2013, but recorded a significant increase between 2014 and 2019 for males. The Malaysian suicide rate of 5.8 per 100,000 population in 2019 was the second highest compared to selected Muslim majority countries, in the middle range compared to other ASEAN countries, and lower than all G7 countries except Italy. However, due to the limitations to the data quality, we could not firmly conclude that the rates and trends reflected the true number of suicide deaths in Malaysia. High quality surveillance of suicidal behavior, both fatal and non-fatal, is essential for identifying the at-risk populations so that appropriate and effective interventions can be tailored and targeted to mitigate their suicidal behavior and ideation.

## Data Availability Statement

Publicly available datasets were analyzed in this study. This data can be found here: the datasets analyzed for this study can be found in the WHO Global Health Estimates: Suicide Rates (WHO Global Health Observatory, 2021) https://www.who.int/data/gho/data/themes/mental-health/suicide-rates.

## Author Contributions

BL, KK, and DL conceptualized the study. BL, KK, CS, and WC extracted and analyzed the data. All authors wrote the first draft of the manuscript, commented on the manuscript, and contributed to and approved the final manuscript.

## Funding

This study was supported by a grant from Geran Galakan Penyelidik Muda, Universiti Kebangsaan Malaysia (GGPM-2021-031).

## Conflict of Interest

The authors declare that the research was conducted in the absence of any commercial or financial relationships that could be construed as a potential conflict of interest.

## Publisher's Note

All claims expressed in this article are solely those of the authors and do not necessarily represent those of their affiliated organizations, or those of the publisher, the editors and the reviewers. Any product that may be evaluated in this article, or claim that may be made by its manufacturer, is not guaranteed or endorsed by the publisher.
